# Supplementary material for: Ocular Paraneoplastic Syndromes
Source: Biomedicines. 2020 Nov 10;8(11):490. doi: 10.3390/biomedicines8110490 (PMC7698240; doi:10.3390/biomedicines8110490)
Supplement: Supplementary file 1 [file biomedicines-08-00490-s001.pdf]

Supplementary materials

Search strategy overview

PubMed

(“ocular paraneoplastic syndrome”[MeSHTerms]) OR (“cancer-associated retinopathy” [MeSHTerms]) OR(“cancer-associated cone dysfunction” [MeSHTerms]) OR (“melanoma-associated retinopathy” [MeSHTerms]) OR (“bilateral diffuse uveal melanocytic proliferation”[MeSHTerms]) OR (“paraneoplastic optic neuritis” [MeSHTerms]) OR (“paraneoplastic vitelliform maculopathy” [MeSH Terms]) OR (“acute exudative paraneoplastic polymorphous vitelliform maculopathy” [MeSH Terms])

AND((Meta-analysis OR “meta analysis” OR metaanalysis OR “systematic review”[Title/Abstract])OR (systematic review OR PT meta-analysis[Publication Type]))

Embase (Elsevier)

‘ocular paraneoplastic syndrome’ OR ‘cancer-associated retinopathy’ OR‘cancer-associated cone dysfunction’ OR ‘melanoma-associated retinopathy’ OR ‘bilateral diffuse uveal melanocytic proliferation’ OR ‘paraneoplastic optic neuritis’ OR ‘paraneoplastic vitelliform maculopathy’ OR ‘acute exudative paraneoplastic polymorphous vitelliform maculopathy’ OR

AND'meta analysis':ab,ti OR metaanalysis:ab,ti OR 'systematic review':ab,ti OR 'systematic review':it OR 'meta analysis':itAND [embase]/lim NOT ([embase]/lim AND [medline]/lim)

Latin American and Caribbean Center on Health Sciences Information(LILACS)

MH: (“ocular paraneoplastic syndrome”) OR (“cancer-associated retinopathy”) OR (“cancer-associated cone dysfunction”) OR (“melanoma-associated retinopathy”) OR (“bilateral diffuse uveal melanocytic proliferation”) OR (“paraneoplastic optic neuritis”) OR (“paraneoplastic vitelliform maculopathy”) OR (“acute exudative paraneoplastic polymorphous vitelliform maculopathy”)

AND (“systematic review” OR "estudosistematico" OR "meta-analise" OR "meta-analisis" OR metaanalise OR metaanalisis)

Cochrane CENTRAL

(MeSH descriptor: [ocular paraneoplastic syndrome] explode all trees OR MeSH descriptor: [cancer-associated retinopathy] explode all trees OR MeSH descriptor: [cancer-associated cone dysfunction] explode all trees OR MeSH descriptor:[melanoma-associated retinopathy] explode all trees OR MeSH descriptor:[bilateral diffuse uveal melanocytic proliferation] explode all trees OR MeSH descriptor:[paraneoplastic optic neuritis] explode all trees OR MeSH descriptor:[paraneoplastic vitelliform maculopathy] explode all trees OR paraneoplastic syndrome* OR cancer-associated retinopathy* OR cancer-associated cone dysfunction* OR melanoma-assiciated retinopathy* OR bilateral diffuse uveal melanocytic proliferation* OR paraneoplastic optic neuritis* OR paraneoplastic vitelliform maculopathy* OR acute exudative paraneoplastic polymorphous vitelliform maculopathy*)

Web of Science

TS =(“ocular paraneoplastic syndrome”) OR (“cancer-associated retinopathy”) OR (“cancer-associated cone dysfunction”) OR (“melanoma-associated retinopathy”) OR (“bilateral diffuse uveal melanocytic proliferation”) OR (“paraneoplastic optic neuritis”) OR (“paraneoplastic vitelliform maculopathy”) OR (“acute exudative paraneoplastic polymorphous vitelliform maculopathy”) OR

Databases= WOS, BIOSIS, CABI, FSTA, KJD, MEDLINE, RSCI, SCIELO, ZOOREC Timespan=All yearsSearch language=Auto AND TS=("systematic review" OR meta-analysis OR "meta analysis" OR metaanalysis)Databases= WOS, BIOSIS, CABI, FSTA, KJD, MEDLINE, RSCI, SCIELO, ZOOREC Timespan=All years

Search language=Auto

Epistemonikos

(((title:((“ acute exudative paraneoplastic polymorphous vitelliform maculopathy “ OR “ocular paraneoplastic syndrome” OR “cancer-associated retinopathy” OR “cancer-associated cone dysfunction” OR “melanoma-associated retinopathy” OR “bilateral diffuse uveal melanocytic proliferation” OR “paraneoplastic optic neuritis” OR “paraneoplastic vitelliform maculopathy”)) OR abstract:((“ acute exudative paraneoplastic polymorphous vitelliform maculopathy “ OR “ocular paraneoplastic syndrome” OR “cancer-associated retinopathy” OR “cancer-associated cone dysfunction” OR “melanoma-associated retinopathy” OR “bilateral diffuse uveal melanocytic proliferation” OR “paraneoplastic optic neuritis” OR “paraneoplastic vitelliform maculopathy”))) AND (title:(Meta-analysis OR "meta analysis" OR metaanalysis OR "systematic review") OR abstract:(Meta-analysis OR "meta analysis" OR metaanalysis OR "systematic review"))) OR abstract:((title: ((“ acute exudative paraneoplastic polymorphous vitelliform maculopathy “OR “ocular paraneoplastic syndrome” OR “cancer-associated retinopathy” OR “cancer-associated cone dysfunction” OR “melanoma-associated retinopathy” OR “bilateral diffuse uveal melanocytic proliferation” OR “paraneoplastic optic neuritis” OR “paraneoplastic vitelliform maculopathy”)) OR abstract: ((“ acute exudative paraneoplastic polymorphous vitelliform maculopathy “ OR “ocular paraneoplastic syndrome” OR “cancer-associated retinopathy” OR “cancer-associated cone dysfunction” OR “melanoma-associated retinopathy” OR “bilateral diffuse uveal melanocytic proliferation” OR “paraneoplastic optic neuritis” OR “paraneoplastic vitelliform maculopathy”))) AND (title:(Meta-analysis OR "meta analysis" OR metaanalysis OR "systematic review") OR abstract:(Meta-analysis OR "meta analysis" OR metaanalysis OR "systematic review"))))

PDQ Evidence

“ocular paraneoplastic syndrome” OR “cancer-associated retinopathy” OR “cancer-associated cone dysfunction” OR “melanoma-associated retinopathy” OR “bilateral diffuse uveal melanocytic proliferation” OR “paraneoplastic optic neuritis” OR “paraneoplastic vitelliform maculopathy” OR “acute exudative paraneoplastic polymorphous vitelliform maculopathy”
